# Multi-dimensional characterization of electrostatic surface potential computation on graphics processors

**DOI:** 10.1186/1471-2105-13-S5-S4

**Published:** 2012-04-12

**Authors:** Mayank Daga, Wu-chun Feng

**Affiliations:** 1 Department of Computer Science, Virginia Tech, Blacksburg, VA 24060, USA; 2 Department of Electrical and Computer Engineering, Virginia Tech, Blacksburg, VA 24061, USA; 3 Virginia Bioinformatics Institute, Virginia Tech, Blacksburg, VA 24061, USA

## Abstract

**Background:**

Calculating the *electrostatic surface potential (ESP) *of a biomolecule is critical towards understanding biomolecular function. Because of its quadratic computational complexity (as a function of the number of atoms in a molecule), there have been continual efforts to reduce its complexity either by improving the algorithm or the underlying hardware on which the calculations are performed.

**Results:**

We present the combined effect of (i) a multi-scale approximation algorithm, known as *hierarchical charge partitioning (HCP)*, when applied to the calculation of ESP and (ii) its mapping onto a graphics processing unit (GPU). To date, most molecular modeling algorithms perform an artificial partitioning of biomolecules into a grid/lattice on the GPU. In contrast, HCP takes advantage of the natural partitioning in biomolecules, which in turn, better facilitates its mapping onto the GPU. Specifically, we characterize the effect of known GPU optimization techniques like use of shared memory. In addition, we demonstrate how the cost of divergent branching on a GPU can be amortized across algorithms like HCP in order to deliver a massive performance boon.

**Conclusions:**

We accelerated the calculation of ESP by *25-fold *solely by parallelization on the GPU. Combining GPU and HCP, resulted in a speedup of at most *1,860-fold *for our largest molecular structure. The baseline for these speedups is an implementation that has been *hand-tuned *SSE-optimized and parallelized across 16 cores on the CPU. The use of GPU does not deteriorate the accuracy of our results.

## Background

Electrostatic interactions in a molecule are of utmost importance for analyzing its structure [1-3] as well as functional activities like ligand binding [4], complex formation [5] and proton transport [6]. The calculation of electrostatic interactions continues to be a computational bottleneck primarily because they are long-range by nature of the $\frac{1}{r}$potential [7]. As a consequence, efficient approximation algorithms have been developed to reduce this computational complexity (e.g., the spherical cut-off method [8], the particle mesh Ewald (PME) method [9], the fast multipole method [10] and the hierarchical charge partitioning (HCP) [11]). The approximation algorithms can be parallelized on increasingly ubiquitous multi- and many-core architectures to deliver even greater performance benefits.

Widespread adoption of general-purpose graphics processing units (GPUs) has made them popular as accelerators for parallel programs [12]. The increased popularity has been assisted by (i) phenomenal computing power, (ii) superior performance/dollar ratio, and (iii) compelling performance/watt ratio. For example, an 8-GPU cluster, costing a few thousand dollars, can simulate 52 ns/day of the JAC Benchmark as compared to 46 ns/day on the Kraken supercomputer, housed at Oak Ridge National Lab and which costs *millions *of dollars [13]. The emergence of GPUs as an attractive high-performance computing platform is also evident from the fact that three out of the top five fastest supercomputers on the Top500 list employ GPUs [14].

Although the use of approximation algorithms can improve performance, they often lead to an increase in the memory boundedness of the application. Achieving optimum performance with a memory-bound application is challenging due to the 'memory wall' [15]. The effect of the memory wall is more severe on GPUs because of the extremely high latency for global memory accesses (on the order of 600 - 800 cycles). Furthermore, for maximum performance on the GPU, execution paths on each GPU computational unit need to be synchronized. However, an important class of approximation algorithms, i.e., multi-scale approximations result in highly asynchronous execution paths due to the introduction of a large number divergent branches, which depend upon the relative distances between interacting atoms.

To test these expectations, we present a hybrid approach wherein we implement the robust multi-scale HCP approximation algorithm in a molecular modeling application called GEM [7] and map it onto a GPU. We counteract the high memory boundedness of HCP by explicitly managing the data movement, in a way that helps us achieve significantly improved performance. In addition, we employ the standard GPU optimization techniques, such as coalesced memory accesses and the use of shared memory, quantifying the effectiveness of each optimization in our application. HCP results in supreme performance on the GPU despite the introduction of divergent branches. This is attributed to the reduction in memory transactions that compensates for divergent branching.

Recently, several molecular modeling applications have used the GPU to speed-up electrostatic computations. Rodrigues et al. [16] and Stone et al. [17] demonstrate that the estimation of electrostatic interactions can be accelerated by the use of spherical cut-off method and the GPU. In [18], Hardy et al. used a multi-scale summation method on the GPU. Each of the aforementioned implementations artificially maps the *n *atoms of a molecule onto a *m*-point lattice grid and then applies their respective approximation algorithm. By doing so, they reduce the time complexity of the computation from *O*(*nn*) to *O*(*nm*). In contrast, we use HCP, which performs approximations based on the natural partitioning of biomolecules. The advantage of using the natural partitioning is that even with the movement of atoms during molecular dynamics simulations, the hierarchical nature is preserved, whereas with the lattice, atoms may move in and out of the lattice during the simulation. Our implementation realizes a maximum of *1,860-fold *speedup over a *hand-tuned *SSE optimized implementation on a modern 16-core CPU, without any loss in the accuracy of the results.

## Methods

### Electrostatics and the hierarchical charge partitioning approximation

We use the Analytic Linearized Poisson-Boltzmann (ALPB) model to perform electrostatic computations [19]. Equation (1) computes the electrostatic potential at a surface-point (vertex) of the molecule due to a single point charge, *q*. The potential at each vertex can be computed as the summation of potentials due to all charges in the system. If there are *P *vertices, the total surface potential can then be found as the summation of potential at each vertex.

(1) $$\phi_{i}^{outside}=\frac{q_{i}}{\varepsilon_{in}}\frac{1}{\left(1+\alpha \frac{\varepsilon_{in}}{\varepsilon_{out}}\right)}\left[\frac{1+\alpha }{d_{i}}-\frac{\alpha \left(1-\frac{\varepsilon_{in}}{\varepsilon_{out}}\right)}{r}\right]$$

Computing the potential at *P *vertices results in a time complexity of *O*(*NP*) where *N *is the number of atoms in the molecule. To reduce the time complexity, we apply an approximation algorithm called hierarchical charge partitioning (HCP), which reduces the upper bound of computation to *O*(*P *log *N*).

**Figure 1:**
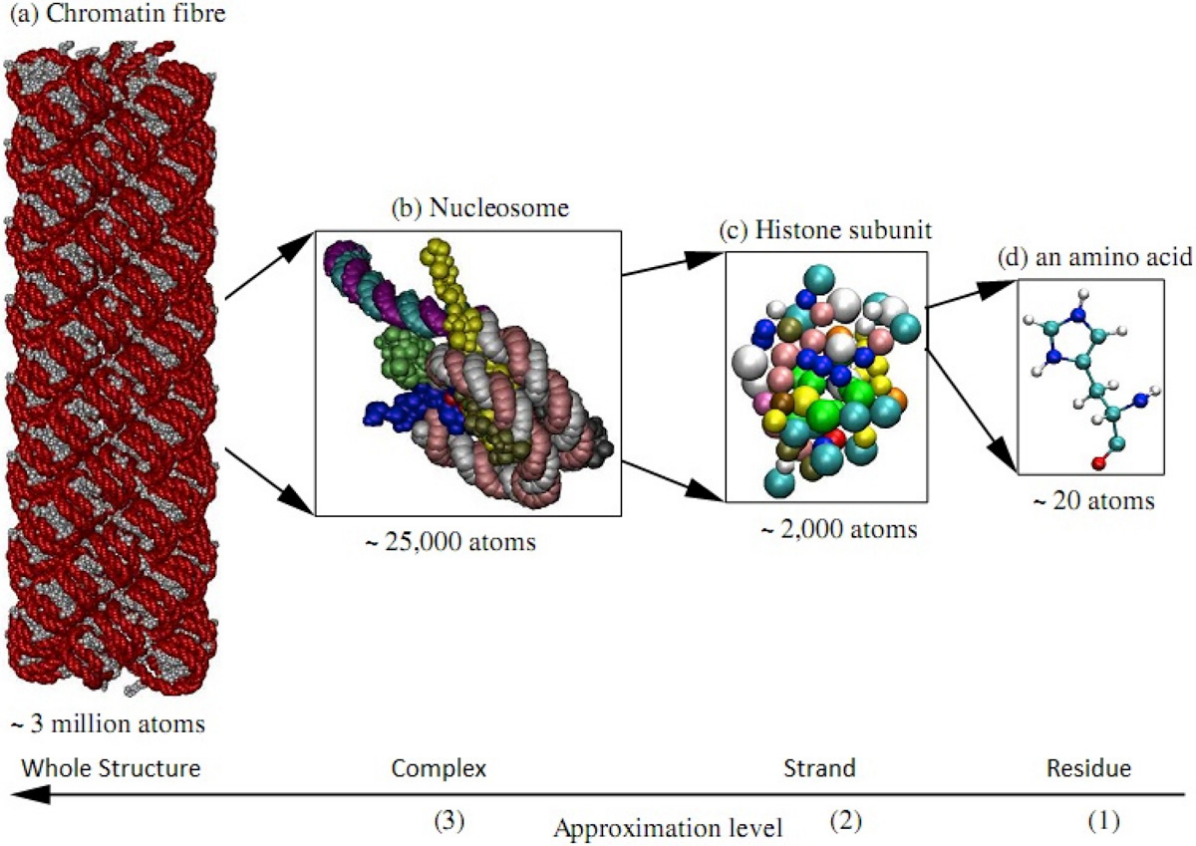
**Illustration of the hierarchical charge partitioning (HCP) of biomolecular structures**. In this illustration a biomolecular structure is partitioned into multiple hierarchical levels components based on the natural organization of biomolecules. The charge distribution of distant components are approximated by a small number of charges, while nearby atoms are treated exactly.

**Figure 2:**
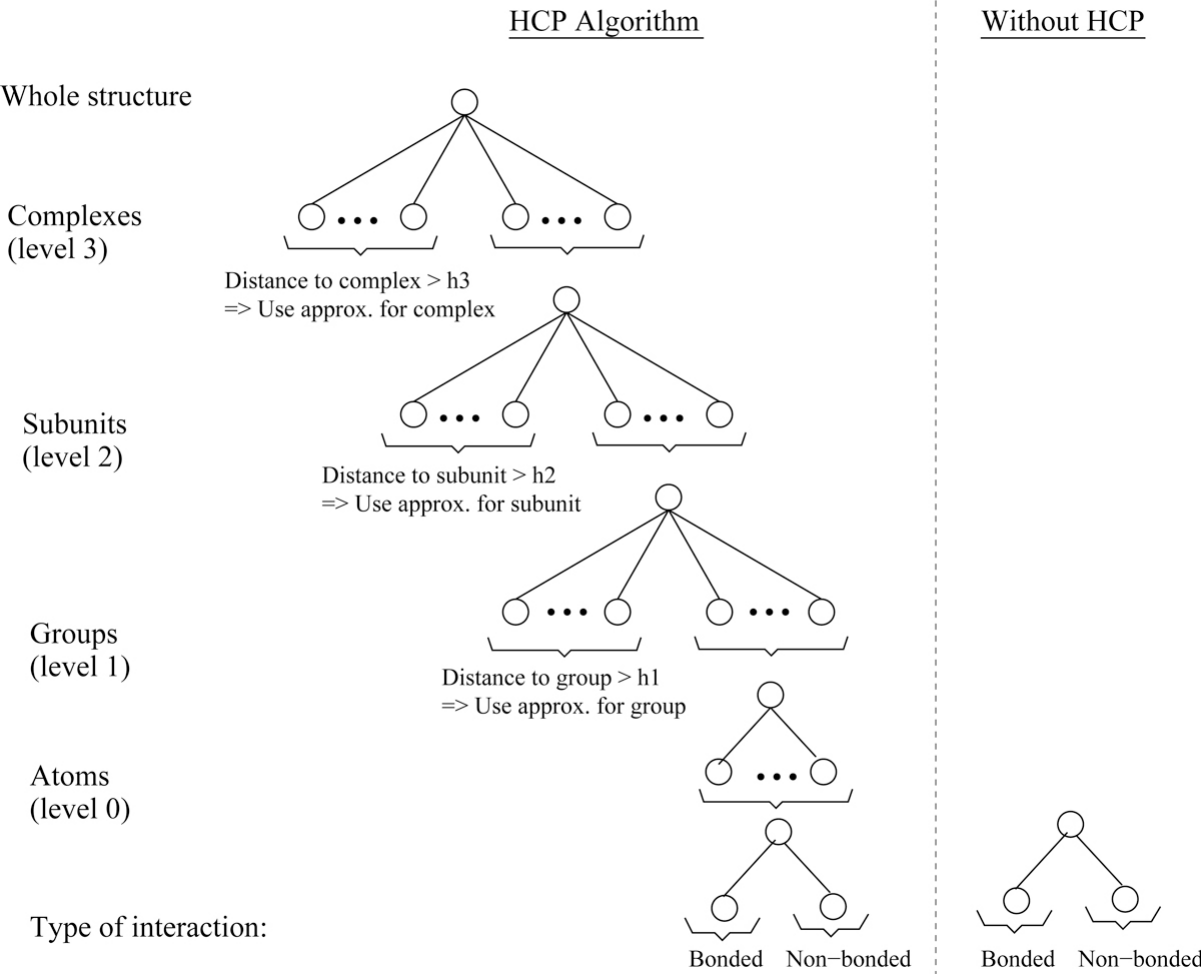
**Illustration of the HCP multi-scale algorithm**. Predefined threshold distances (*h*_1_, *h*_2_, *h*_3_) are used to determine the level of approximation used in the HCP approximation. This top-down algorithm results in ~ *NLogN *scaling compared to a ~ *N*^2 ^scaling without HCP.

HCP [11] exploits the natural partitioning of biomolecules into constituent structural components in order to speed-up the computation of electrostatic interactions with limited and controllable impact on accuracy. Biomolecules can be systematically partitioned into multiple molecular *complexes*, which consist of multiple polymer chains or *subunits *and which in turn are made up of multiple amino acid or nucleotide *groups*, as illustrated in Figure 1. Atoms represent the lowest level in the hierarchy while the highest level depends on the problem. Briefly, HCP works as follows. The charge distribution of components, other than at the atomic level, is approximated by a small set of point charges. The electrostatic effect of distant components is calculated using the smaller set of point charges, while the full set of atomic charges is used for computing electrostatic interactions within nearby components. The distribution of charges for each component, used in the computation, varies depending on distance from the point in question: the farther away the component, the fewer charges are used to represent the component. The actual speedup from using HCP depends on the specific hierarchical organization of the biomolecular structure as that would govern the number of memory accesses, computations and divergent branches on the GPU. Under conditions consistent with the hierarchical organization of realistic biomolecular structures, the top-down HCP algorithm (Figure 2) scales as *O*(*N *log *N*), where *N *is the number of atoms in the structure. For large structures, the HCP can be several orders of magnitude faster than the exact *O*(*N*^2^) all-atom computation. A detailed description of the HCP algorithm can be found in Anandakrishnan et. al. [11].

### GPU architecture and programming interface

For this study, we have used state-of-art NVIDIA GPUs based on the Compute Unified Device Architecture or CUDA framework. CUDA is a framework developed by NVIDIA, which facilitates the implementation of general-purpose applications on GPUs. Below is a brief description of the NVIDIA GPU hardware architecture and the CUDA programming interface.

**Figure 3:**
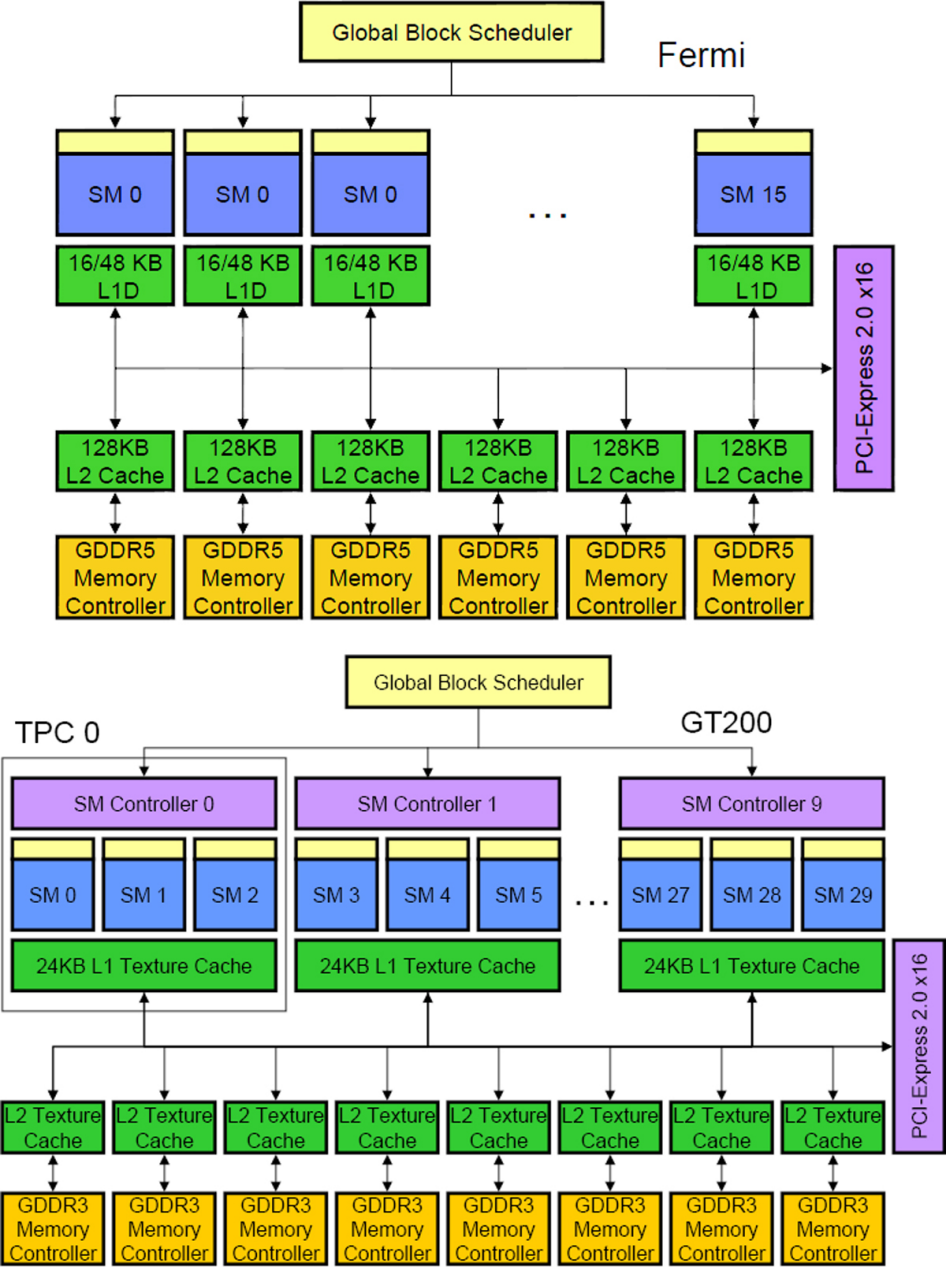
**Overview of NVIDIA GPU architectures**. The Fermi architecture is shown to consist of 16 Streaming Multiprocessors (SMs). Each SM consists of 64 KB of on-chip memory, which can configured as 16 KB of shared memory and 48 KB of L1 data cache or vice versa. Also present on each SM is 128 KB of L2 data cache. The GDDR5 memory controllers facilitate data accesses to and from the global memory. The GT200 architecture consists of 30 SMs. Each SM consists of 16 KB of shared memory but no data caches, instead it contains L1/L2 texture memory space. Also present are GDDR3 memory controllers to facilitate global memory accesses.

NVIDIA GPUs consist of 240-512 execution units, which are grouped into 16 and 30 streaming multiprocessors (SMs) on Fermi and GT200 architectures, respectively. An overview of these architectures is shown in Figure 3. Multiple threads on a GPU execute the same instruction, resulting in a single instruction, multiple thread (SIMT) architecture. This is what makes GPU very suitable for applications that exhibit data parallelism, i.e., the operation on one data element is independent of the operations on other data elements. Therefore, it is well suited for molecular modeling where the potential at one vertex can be computed independently of all others.

On NVIDIA GPUs, threads are organized into groups of 32, referred to as a *warp*. When threads within a warp follow different execution paths, such as when encountering a conditional, a divergent branch takes place. Execution of these group of threads is serialized, thereby, affecting performance. On a GPU, computations are much faster compared to a typical CPU, but memory accesses and divergent branching instructions are slower. The effect of slower memory access and divergent branching can be mitigated by initiating thousands of threads on a GPU, such that when one of the threads is waiting on a memory access, other threads can perform meaningful computations.

Every GPU operates in a memory space known as global memory. Data which needs to be operated on by the GPU, needs to be first transferred to the GPU. This process of transferring data to GPU memory is performed over the PCI-e bus, making it an extremely slow process. Therefore, memory transfers should be kept to a minimum to obtain optimum performance. Also, accessing data from the GPU global memory entails the cost of 400-600 cycles and hence, *on-chip *memory should be used to reduce global memory traffic. On the GT200 architecture, each SM contains a high-speed, 16 KB, scratch-pad memory, known as the shared memory. Shared memory enables extensive re-use of data, thereby, reducing *off-chip *traffic. Whereas on the latest Fermi architecture, each SM contains 64 KB of *on-chip *memory, which can be either be configured as 16 KB of shared memory and 48 KB of L1 cache or vice versa. Each SM also consists of a L2 cache of size 128 KB. The hierarchy of caches on the Fermi architecture allows for more efficient global memory access patterns.

CUDA provides a C/C++ language extension with application programming interfaces (APIs). A CUDA program is executed by a *kernel*, which is effectively a function call to the GPU, launched from the CPU. CUDA logically arranges the threads into blocks which are in turn grouped into a grid. Each thread has its own ID which provides for one-one mapping. Each block of threads is executed on a SM and share data using the shared memory present.

### Mapping HCP onto GPU

The problem of computing molecular surface potential is inherently data parallel in nature, i.e., the potential at one point on the surface can be computed independently from the computation of potential at some other point. This works to our advantage as such applications map very well onto the GPU. We begin with offloading all the necessary data (coordinates of vertices and atoms and the approximated point charges) to the GPU global memory. To ensure efficient global memory accesses patterns, we flattened the data structures. By flattening of data structures we mean that all the arrays of structures were transformed into arrays of primitives so that the threads in a half warp (16 threads) access data from contiguous memory locations [20,21]. The GPU kernel is then executed, wherein each thread is assigned the task of computing the electrostatic potential at one vertex. At this point the required amount of shared memory, i.e, number of threads in a block times the size of the coordinates of each vertex, is allocated on each streaming multiprocessor (SM) of the GPU. The kernel is launched multiple times as required, until all the vertices are exhausted, with implicit GPU synchronization in between successive kernel launches. On the GPU side, each kernel thread copies the coordinates of its assigned vertex onto the shared memory. This results in a reduction of the number of global memory loads as explained in the Results section. The limited amount of per SM shared memory does not allow us to offload the coordinates of constituent components of the biomolecule and hence, coordinates of complexes, strands, residues, and atoms have to remain in global memory. The HCP algorithm is then applied to compute the electrostatic potential, and the result is stored in the global memory. All the threads perform this computation in parallel, and after the threads finish, the computed potential at each vertex is transferred back to the CPU memory, where a reduce (sum) operation is performed to calculate the total molecular surface potential. According to the algorithm, evaluation of distance between the vertex and molecular components requires each thread to accesses coordinates from the global memory. This implies that potential calculation at each vertex necessitates multiple global memory accesses, which makes HCP memory-bound on the GPU.

HCP also introduces a significant number of divergent branches on the GPU. This phenomenon occurs because for some threads in a warp, it may be possible to apply HCP approximation while for other, it may not be possible to do so. Therefore, these two groups of threads would diverge and follow respective paths, resulting in a divergent branch. In the Results section, we show how the associated costs of divergent branching in HCP on the GPU can be amortized to deliver a performance boost.

### Test setup

To illustrate the scalability of our application, we have used four different structures with varied sizes. The characteristics of the structures used are presented in Table 1. The GPU implementation was tested on the present generation of NVIDIA GPUs.

**Table 1:** Characteristics of input structures

Structure	#Vertices	#Complexes	#Strands	#Residues	#Atoms
H helix myoglobin, 1MBO	5,884	1	1	24	382
nuclesome core particle, 1KX5	258,797	1	10	1,268	25,086
chaperonin GroEL, 2EU1	898,584	1	14	7,336	109,802
virus capsid, 1A6C	593,615	1	60	30,780	476,040

The Host Machine consists of an E8200 Intel Quad core running at 2.33 GHz with 4 GB DDR2 SDRAM. The operating system on the host is a 64-bit version of Ubuntu 9.04 distribution running the 2.6.28-16 generic Linux kernel. Programming and access to the GPU was provided by CUDA 3.1 toolkit and SDK with the NVIDIA driver version 256.40. For the sake of accuracy of results, all the processes that required a graphical user interface were disabled to limit resource sharing of the GPU.

We ran our tests on a NVIDIA Tesla C1060 graphics card with GT200 GPU and the NVIDIA Fermi Tesla C2050 graphics card. An overview of both of these GPUs is presented in Table 2.

**Table 2:** Overview of GPUs used

GPU	Tesla C1060	Fermi Tesla C2050
Streaming Processor Cores	240	448
Streaming Multiprocessors (SMs)	30	16
Memory Bus type	GDDR3	GDDR5
Device Memory size	4096 MB	3072 MB
Shared Memory (per SM)	16 KB	Configurable 48 KB or 16 KB
L1 Cache (per SM)	None	Configurable 16KB or 48 KB
L2 Cache	None	768 KB
Double Precision Floating Point Capability	30 FMA ops/clock	256 FMA ops/clock
Single Precision Floating Point Capability	240 FMA ops/clock	512 FMA ops/clock
Special Function Units (per SM)	2	4
Compute Capability	1.3	2.0

## Results and discussion

In this section, we present an analysis of (i) the impact of using shared memory, (ii) the impact of divergent branching, (iii) the speedups realized by our implementation, and (iv) the accuracy of our results. On CPU, the timing information was gathered by placing required time-checking calls around the computational kernel, excluding the I/O required for writing the results. On GPU, the execution time was measured by using the CUDAEventRecord function call. For a fair comparison, time for offloading the data onto the GPU global memory and storing the results back onto the CPU was taken into account along with the time for execution of the kernel. Single precision was used on both platforms. All the numbers presented are an average of 10 runs performed on each platform. For HCP, the 1st-level threshold was set to 10Å and the 2nd-level threshold was fixed at 70Å.

### Impact of using shared memory

At every approximation level, HCP reuses the vertex coordinates to compute the distance between the vertex and molecule components. Therefore in the worst case when no approximation can be applied, same data is accessed *four times *from the global memory (due to four levels in the molecule hierarchy). We used the shared memory to reduce these global memory accesses. Percentage reduction in the number of global memory loads due to the use of shared memory on GT200 architecture, with and without HCP approximation, is shown in Table 3. The base line for each of these columns is the respective implementation, i.e., without_HCP and HCP, without the use of shared memory. These numbers were taken from the CUDA Visual Profiler provided by NVIDIA [22].

**Table 3:** Percentage reduction in the number of global memory loads

Structure	Without HCP	With HCP
H Helix myoglobin	50%	32%
nucleosome core paricle	50%	62%
chaperonin GroEL	50%	84%
virus capsid	50%	96%

From the table, we note that the global memory loads are reduced by 50% for all structures, when the HCP approximation is not used. Whereas with HCP, the amount by which loads are reduced varies from structure to structure. This can be reasoned as follows. When no approximation is applied, the coordinates of vertices and that of all atoms are accessed from global memory, which requires cycling through the residue groups. Therefore when shared memory is not used, the vertex coordinate is loaded twice, once for residue and once for the atom. While when shared memory is used, it is loaded only once, i.e., for copying into the shared memory, thereby, resulting in a 50% reduction in global memory loads.

But in the case of HCP, the number of times a vertex coordinate is loaded from global memory depends upon the structure. This is because for each structure the effective number of computations to be performed are different. For example, for a structure with 1st level of approximation and no shared memory usage, vertex coordinates would be loaded three times from the global memory - (i) to compute the distance to the complex, (ii) to compute the distance to the strand and (iii) finally to compute the distance to the residue. While with shared memory it would be accessed just once. Similarly, for a structure with no approximation, the vertex would be accessed four times, without using shared memory. Therefore, the table suggests that least number of components could be approximated for the virus capsid, and hence, maximum percentage reduction.

Use of the shared memory resulted in a drastic reduction in the number of global loads and hence, provided about 2.7-fold speedup to our application.

### Impact of divergent branching

**Figure 4:**
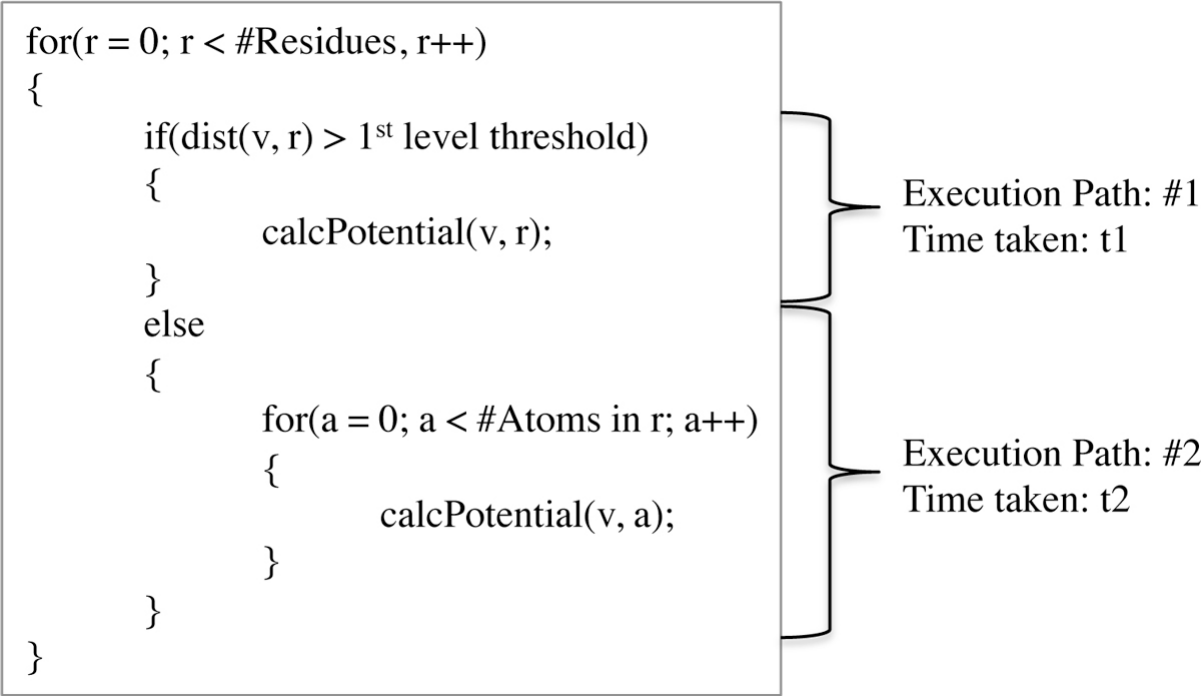
**Divergent branching due to the HCP approximation**. This illustration shows that our GPU implementation causes divergent branches to occur. This is because for every thread, there are probable execution paths. Divergent branches occur if threads within a warp take different paths.

Divergent branching on a GPU occurs when the threads of a warp follow different execution paths. In our GPU implementation, each thread takes charge of one vertex and as shown in Figure 4, it is possible for threads within a warp to follow different execution paths, thereby, introducing a divergent branch. Here we quantify the cost of divergent branches that are introduced. For the ease of exposition, we limited our analysis to one level of HCP only, but this can be extended. As the execution paths are now serialized, the time taken for the execution of a divergent branch, denoted by *t_divBranch _*can be characterized as follows:

(2) $$t_{divBranch}=t_{1}+t_{2}$$

where *t_i _*denotes time taken for execution path *i*, as shown in Figure 4. From the figure, it can be noted that both execution paths perform the similar task of calculating the potential. Path#1 calls the function, calcPotential () just once while for Path#2, calcPotential () is called from within a loop that iterates over all atoms of that residue. Hence, it can be inferred that time for divergent branches is directly proportional to the number of atoms in the molecule.

(3) $$\begin{array}{c} t_{2}\\ ∴t_{divBranch}\\ ∴t_{divBranch} \end{array}\begin{array}{c} \gg \\ \approx \\ \approx \\ \propto \end{array}\begin{array}{c} t_{1}\\ t_{2}\\ \#Atoms\times T_{calcPotential}\\ \#Atoms \end{array}$$

Thus, Total time for all the divergent branches in the system:

(4) $$\begin{array}{ccc} T_{divBranch} & = & \#DivBranches\times t_{divBranch}\\ ∴T_{divBranch} & \propto & \#Atoms \end{array}$$

**Figure 5:**
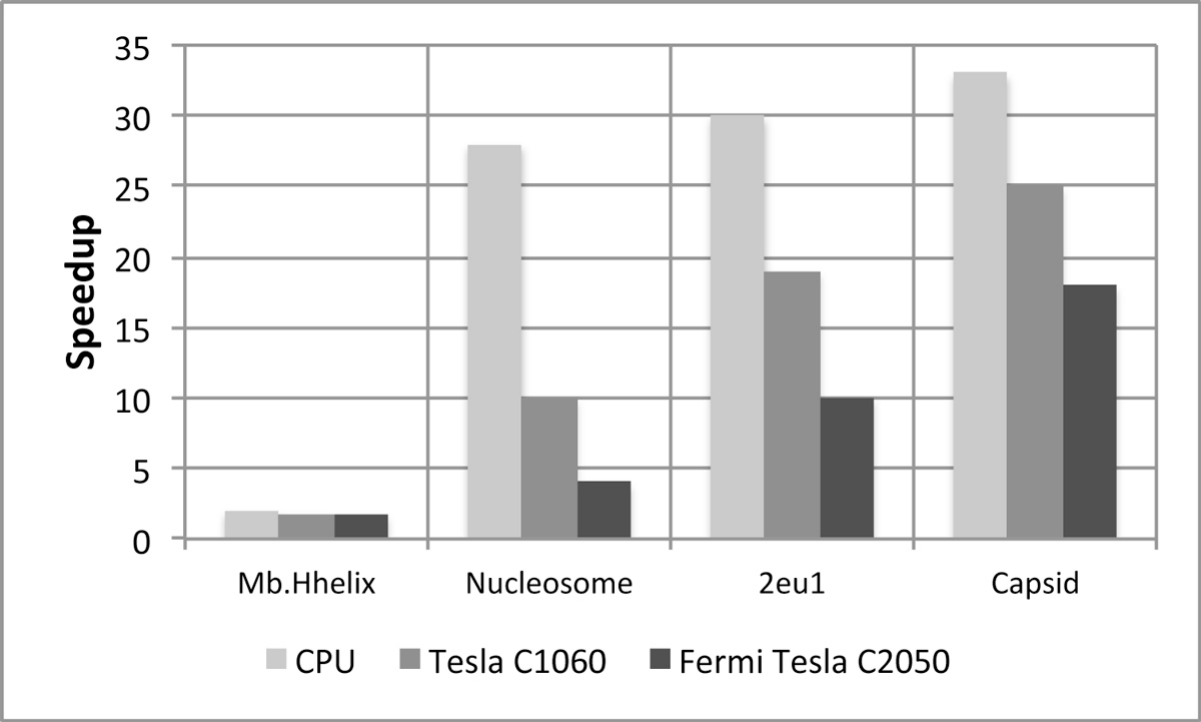
**Speedup due to the HCP approximation**. Even with the occurrence of divergent branches, speedup due to the HCP approximation is positive on the GPU. This alludes to the fact there is some aspect with amortizes the cost of the introduction of these divergent branches. Speedup is maximum for the largest structure, i.e., virus capsid. Baseline: Corresponding implementation on each platform without the use of HCP approximation.

From (4), we can say that the cost of divergent branches would be maximum for the molecule which has the greatest #atoms but this is not true as can be seen from Figure 5. In the figure, we present the speedups achieved due to HCP on CPU as well as the GPUs with #atoms increasing from left to right. As all the speedups are positive, we can safely infer that GPU is indeed beneficial for HCP despite the introduction of divergent branches. We also note that speedup achieved due to HCP on GPUs, increases with the increase in #atoms in the structure, thus, dissatisfying (4). Hence, there exists an aspect which compensates for the cost of introduction of divergent branches. As HCP is a memory-bound application, number of memory transactions dominate the execution time. Looking back at the algorithm (in the Methods section), we observe that HCP does reduce the number of memory transactions. It does so by applying the approximation, which results in reduced fetching of coordinates from the global memory. Now, coordinates of only the higher level component are required and hence, compensating for the cost of divergent branches. Execution time for the entire application with HCP can be derived as follows:

(5) $$\begin{array}{c} {T_{With}}_{\text{\_}HCP}\\ \text{where}\;T_{Mem} \end{array}\begin{array}{c} =T_{Without\text{\_}HCP}T_{Mem}+T_{divBranch}\\ =\text{global memory access time} \end{array}$$

**Figure 6:**
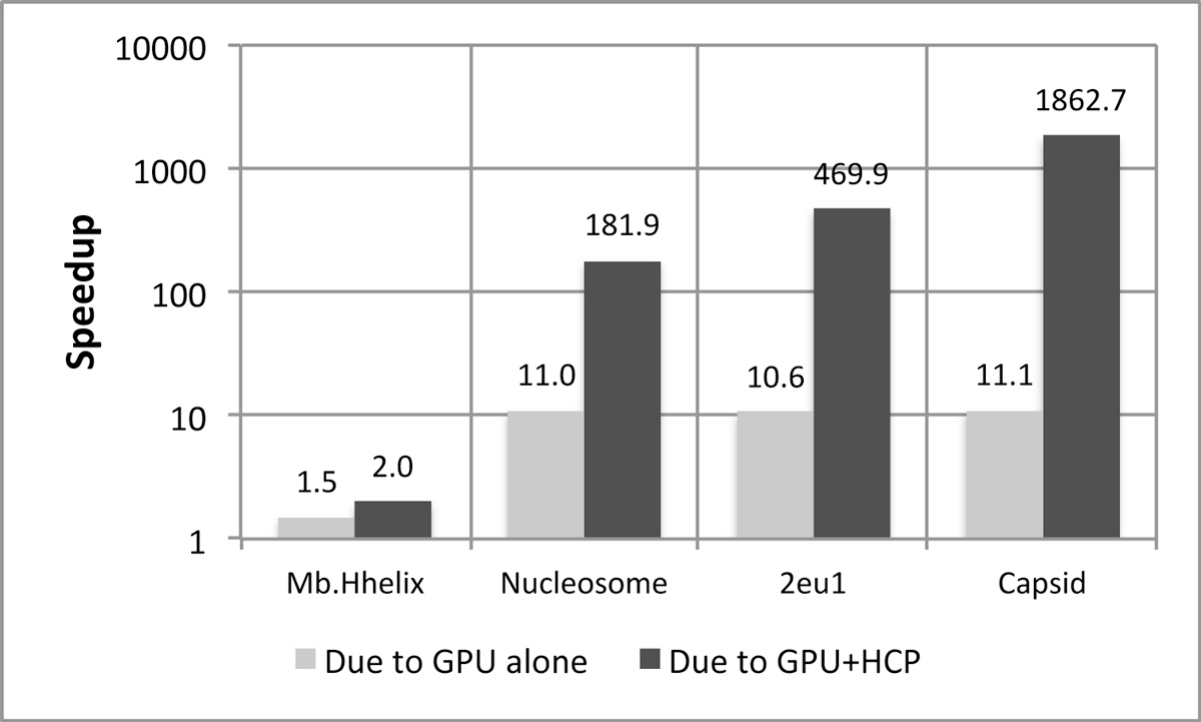
**Speedup on NVIDIA Tesla C1060**. Speedup due the GPU alone is almost constant because once the threshold for the number of threads that can be launched is met, there is no further increase in speedup. Speedup due to HCP+GPU increases with the increase in the size of the structure due to the O(nlogn) scaling of the HCP approximation. Baseline: No-approximation CPU implementation optimized by hand-tuned SSE Intrinsics and parallelized across 16 cores.

**Figure 7:**
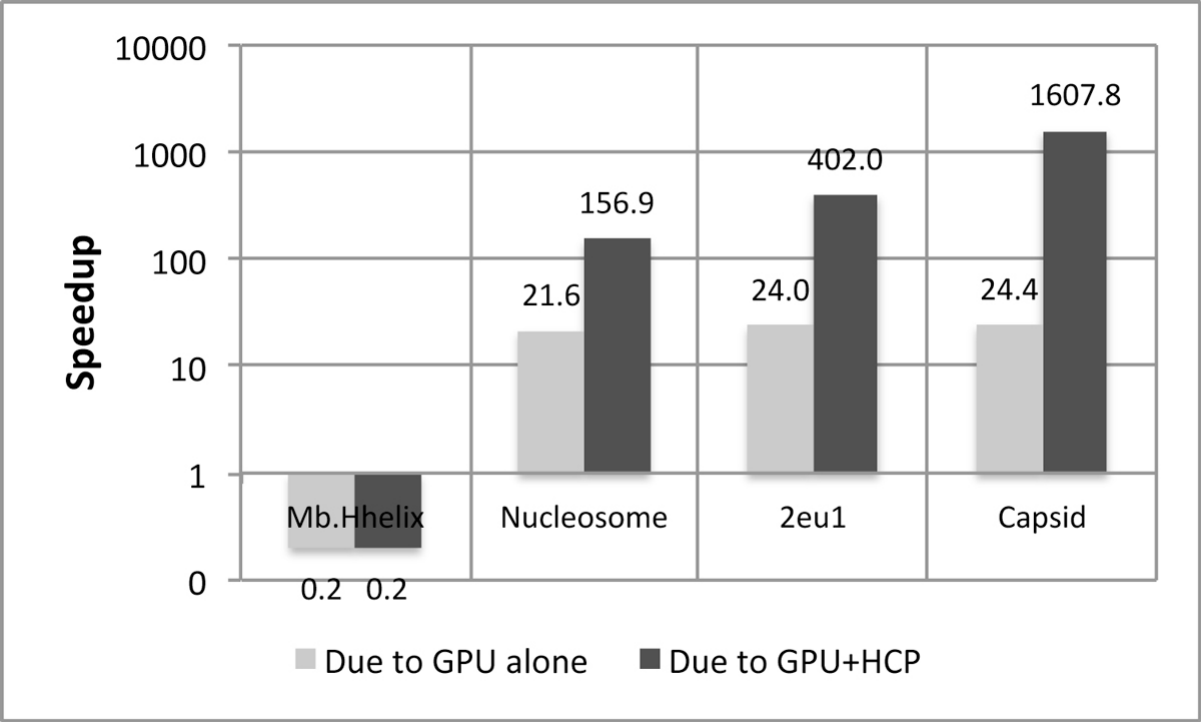
**Speedup on NVIDIA Tesla Fermi C2050**. Speedup on the Tesla Fermi C2050 is greater than Tesla C1060 due to the presence of hierarchy of caches on the C2050 GPU. Baseline: No-approximation CPU implementation optimized by hand-tuned SSE Intrinsics and parallelized across 16 cores.

HCP would guarantee improved performance on the GPU if the gain in time due to reduced memory transactions is greater than the cost of divergent branches. However, computing memory access times on the GPU is an extremely difficult task as one has no knowledge of how warps are scheduled, which is essential as the warps send access requests to memory controllers. Hence, no direct method to measure global memory access times exists. We used an indirect approach and found out the reduction in memory transactions as well as the increase in divergent branches for our application. These numbers have been taken using from the CUDA Visual Profiler provided by NVIDIA and are presented in Table 4[22]. The memory transactions in the table portray the sum of 32-, 64- and 128-byte load and store transactions per SM. Also, the number of divergent branches represent the divergent branches introduced on one SM. From the table, it is seen that the reduction in memory transactions is orders of magnitude greater than the increase in divergent branches. From the table, we note that the number of memory transactions reduced per one divergent branch is maximum for the capsid, which results in the fact that HCP+GPU is most effective for capsid. Figures 6 and 7 corroborate this fact and hence, we can attest that it is the reduction in memory transactions which help make GPUs favorable for HCP. This proves that even an algorithm with divergent branching can be benefited by the GPU, provided there is some aspect which amortizes the cost of the divergent branches introduced.

**Table 4:** Impact of the HCP approximation on memory transactions and divergent branches

Structure	Decrease in # of mem. Transactions	Increase in # of divergent branches
H Helix myoglobin	95,800	34
nucleosome core particle	119,507,436	4,635
chaperonin GroEL	1,831,793,578	25,730
virus capsid	5,321,785,506	22,651

### Speedup

Figures 6 and 7 present speedups achieved by our implementation on NVIDIA Tesla C1060 and NVIDIA Fermi Tesla C2050 GPUs respectively. Both the figures present speedup over the CPU implementation optimized by *hand-tuned *SSE Intrinsics and parallelized across 16 cores, without the use of any approximation algorithm. Speedups achieved due to the use of GPU alone as well as that due to the combination of GPU and HCP are presented for all four structures.

From both these figures, we note that speedup due to the GPU alone is almost constant for all three structures barring Mb.Hhelix. This is because Mb.Hhelix is an extremely small structure and it does not requires enough GPU threads for the computation of its molecular surface potential, thereby, leaving the GPU under utilized. This phenomenon is prominent in case of the Fermi Tesla C2050 which actually results in a slowdown due to under-utilization of the GPU. For other structures the threshold of the number of threads is met and almost similar speedup is achieved across both the figures. The observed speedup is around *11-fold *on Tesla C1060, whereas on Tesla C2050 the speedup is around *25-fold*. The increased speedup on C2050 may be attributed to several architectural differences between Fermi and GT200 GPUs, like the ability for concurrent kernel execution, ECC support and fewer SMs. However, the architectural feature that we feel has the most impact for this algorithm, is the presence of a hierarchy of caches on Fermi, as they allow for greater exploitation of the locality of data. For no approximation, all atoms need to be accessed sequentially, thereby, making the caches play an important role and hence, Fermi Tesla C2050 is deemed to be more effective.

As explained in a previous section, application speedup due to the combination of GPU and HCP increases with the increase in *number of memory transactions reduced per divergent branch increased*. Therefore, from Table 4, number of memory transactions reduced is maximum for virus Capsid and hence, it attains the maximum speedup. Next highest reduction in the number of memory transactions is for 2eu1 and hence, next highest speedup and so on. Our application manages to achieve up to *1,860-fold *speedup with HCP on Tesla C1060 for Capsid while the corresponding speedup on Fermi Tesla C2050 is approximately *1,600-fold*. The actual execution time of our implementation on both GPUs is *<1 sec*.

Speedup achieved with HCP on Tesla C2050 is less than that achieved on Tesla C1060 due to the fact that the algorithm fails to take the advantage of the caches present on Fermi, as before. With HCP, not all memory requests are sequential as coordinates of both atoms and high level components are required, making the caches less potent than before. Speedups achieved across all figures for without_HCP version are almost consistent for both the GPUs, which is because it does not introduce divergent branches. Whereas, the version with HCP, results in divergent branches and also varying amounts of speedups across structures, depending upon how much cost of the divergent branches can be amortized by the corresponding reduction in memory transactions.

### Accuracy

To get the best performance on GPUs, we used single precision as double precision on GT200 architecture adversely impacts the performance by as much as 8-times. Although double precision on Fermi is almost half as fast as single precision, we decided to stick with single precision for greater performance than accuracy. To get an estimate of the accuracy of our results, we compute the relative root mean squared error (RMSE) of the single-precision-GPU implementation against the double-precision-CPU implementation. The results are shown in Table 5. We also present the error due to HCP both on CPU and the GPU. HCP, being an approximation algorithm, does introduce some error on the CPU. From the table, we note that the error introduced by the GPU itself is fairly negligible when compared to the error introduced by HCP alone on the CPU. Thus, the total error due to HCP and GPU is almost equivalent to the error on the CPU. Therefore, we can safely conclude that single precision on GPU does not jeopardize the accuracy of our computed results.

**Table 5:** Relative RMS (root-mean-squared) error

Structure	Version	Relative RMSE
H helix myoglobin	CPU with HCP	0.215821
	GPU	0.000030
	GPU with HCP	0.236093

nuclesome core particle	CPU with HCP	0.022950
	GPU	0.000062
	GPU with HCP	0.022853

chaperonin GroEL, 2eu1	CPU with HCP	0.008799
	GPU	0.000042
	GPU with HCP	0.008816

virus capsid	CPU with HCP	0.015376
	GPU	0.000173
	GPU with HCP	0.015273

Due to the paltry error introduced by single precision on the GPU, it may be deemed acceptable for the computation of molecular surface potential on the GPU but may be unsatisfactory for molecular dynamics. In case of molecular dynamics simulations, even a minute error in one time step can have a substantial effect on the results as the error would accumulate during the course of the simulation. It is here that superior double precision support of Fermi would come in handy.

## Conclusions

With the emergence of GPU computing, there have been many attempts at accelerating the electrostatic surface potential (ESP) computations for biomolecules. In our work, we demonstrate the combined effect of using a multi-scale approximation algorithm called *hierarchical charge partitioning (HCP) *and mapping it onto a graphics processing unit (GPU). While mainstream molecular modeling algorithms impose an artificial partitioning of biomolecules into a grid/lattice to map it onto a GPU, HCP is significantly different in that it takes advantage of the natural partitioning in biomolecules, which facilitates a data-parallel mapping onto the GPU.

We then presented our methodology for mapping and optimizing the performance of HCP on the GPU when applied to the calculation of ESP. Despite being a memory-bound application, we leveraged many known optimization techniques to accelerate performance. In addition, we demonstrated the effectiveness of the introduction of divergent branching on GPUs when it reduces the number of instructional and memory transactions.

For a fairer comparison between the CPU and GPU, we optimized the CPU implementation by using hand-tuned SSE intrinsics to handle the SIMD nature of the application on the CPU. We then demonstrated a *1,860-fold *reduction in the execution time of the application when compared to that of the *hand-tuned *SSE implementation on the 16 cores of the CPU. Furthermore, we ensured that the use of single-precision arithmetic on the GPU, combined with the HCP multi-scale approximation, did not significantly affect the accuracy of our results.

For future work, we will apply our HCP approximation algorithm to molecular dynamics (MD) simulations on the GPU, given how well it performs in the case of molecular modeling. For MD simulations, the use of double precision is mandatory as the error incurred in each time-step would accumulate over time, thereby immensely affecting the accuracy of the MD results. In addition, we plan to exploit the use of the cache hierarchy on the NVIDIA Fermi to accelerate the memory-bounded aspect of our application.

## Competing interests

The authors declare that they have no competing interests.

## Authors' contributions

MD implemented and optimized the HCP approximation on the GPU. MD also studied the impact of divergence and memory transactions on the GPU, collected all the required results and drafted the manuscript. WF conceived the study, co-designed the GPU mapping of HCP and helped draft the manuscript. Both authors read and approved the final manuscript.

## References

[B1] Perutz M Electrostatic effects in proteins Science 1978 201 1187 1191 10.1126/science.694508 694508

[B2] Baker NA McCammon JA Electrostaic Interactions In Structural Bioinformatics 2002 New York: John Wiley & Sons, Inc

[B3] Honig B Nicholls A Classical Electrostatics in Biology and Chemistry Science 1995 268 1144 1149 10.1126/science.7761829 7761829

[B4] Szabo G Eisenman G McLaughlin S Krasne S Ionic probes of membrane structures. In: Membrane Structure and Its Biological Applications Ann NY Acad Sci 1972 195 273 290 10.1111/j.1749-6632.1972.tb54807.x 4504092

[B5] Sheinerman FB Norel R Honig B Electrostatic aspects of protein-protein interactions Curr Opin Struct Biol 2000 10 2 153 9 10.1016/S0959-440X(00)00065-8 10753808

[B6] Onufriev A Smondyrev A Bashford D Proton affinity changes during unidirectional proton transport in the bacteriorhodopsin photocycle J Mol Biol 2003 332 1183 1193 10.1016/S0022-2836(03)00903-3 14499620

[B7] Gordon JC Fenley AT Onufriev A An analytical approach to computing biomolecular electrostatic potential. II. Validation and applications The Journal of Chemical Physics 2008 129 7 075102 10.1063/1.2956499 19044803 PMC2671192

[B8] Ruvinsky AM Vakser IA Interaction cutoff effect on ruggedness of protein-protein energy landscape Proteins: Structure, Function, and Bioinformatics 2008 70 4 1498 1505 10.1002/prot.21644 17910068

[B9] Darden T York D Pedersen L Particle mesh Ewald: An N.log(N) method for Ewald sums in large systems The Journal of Chemical Physics 1993 98 12 10089 10092 10.1063/1.464397

[B10] Cai W Deng S Jacobs D Extending the fast multipole method to charges inside or outside a dielectric sphere J Comp Phys 2006 223 846 864 10.1016/j.jcp.2007.09.001 PMC2219213 18235844

[B11] Anandakrishnan R Onufriev A An *N *log *N *approximation based on the natural organization of biomolecules for speeding up the computation of long range interactions Journal of Computational Chemistry 2010 31 4 691 706 19569183 10.1002/jcc.21357 PMC2818067

[B12] Buck I Foley T Horn D Sugerman J Fatahalian K Houston M Hanrahan P Brook for GPUs: stream computing on graphics hardware International Conference on Computer Graphics and Interactive Techniques 2004 ACM New York, NY, USA 777 786

[B13] Huang JH Opening Keynote, NVIDIA GTC, 2010 2010 http://livesmooth.istreamplanet.com/nvidia100921/

[B14] The Top500 Supercomputer Sites http://www.top500.org

[B15] Asanovic K Bodik R Catanzaro BC Gebis JJ Husbands P Keutzer K Patterson DA Plishker WL Shalf J Williams SW Yelick KA The Landscape of Parallel Computing Research: A View from Berkeley 2006 Tech Rep UCB/EECS-2006-183, EECS Department, University of California, Berkeley http://www.eecs.berkeley.edu/Pubs/TechRpts/2006/EECS-2006-183.html

[B16] Rodrigues CI Hardy DJ Stone JE Schulten K Hwu WMW GPU acceleration of cutoff pair potentials for molecular modeling applications Proceedings of the 5th conference on Computing frontiers, CF '08 2008 New York, NY, USA: ACM 273 282 http://doi.acm.org/10.1145/1366230.1366277

[B17] Stone JE Phillips JC Freddolino PL Hardy DJ Trabuco LG Schulten K Accelerating molecular modeling applications with graphics processors Journal of Computational Chemistry 2007 28 16 2618 2640 10.1002/jcc.20829 17894371

[B18] Hardy DJ Stone JE Schulten K Multilevel summation of electrostatic potentials using graphics processing units Parallel Computing 2009 35 3 164 177 10.1016/j.parco.2008.12.005 20161132 PMC2743154

[B19] Fenley AT Gordon JC Onufriev A An analytical approach to computing biomolecular electrostatic potential. I. Derivation and analysis The Journal of Chemical Physics 2008 129 7 075101 http://scitation.aip.org/getabs/servlet/GetabsServlet?prog=normal\&id=JCPSA6000129000007075101000001\&idtype=cvips\&gifs=yes 10.1063/1.2956497 19044802 PMC2671191

[B20] NVIDIA NVIDIA CUDA Programming Guide-3.2 2010 http://developer.download.nvidia.com/compute/cuda/3_2/toolkit/docs/CUDA_C_Programming_Guide.pdf

[B21] Anandakrishnan R Fenley A Gordon J Feng W Onufriev A Accelerating electrostatic surface potential calculation with multiscale approximation on graphical processing units Journal of Molecular Graphics and Modelling 2009 Submitted 10.1016/j.jmgm.2010.04.001 PMC2907926 20452792

[B22] NVIDIA CUDA Visual Profiler 2009 http://developer.download.nvidia.com/compute/cuda/2_2/toolkit/docs/cudaprof_1.2_readme.html

